# Prediction of Major Bleeding in Anticoagulated Patients for Venous Thromboembolism: Comparison of the RIETE and the VTE-BLEED Scores

**DOI:** 10.1055/s-0041-1729171

**Published:** 2021-08-09

**Authors:** Ramón Lecumberri, Laura Jiménez, Pedro Ruiz-Artacho, José Antonio Nieto, Nuria Ruiz-Giménez, Adriana Visonà, Andris Skride, Fares Moustafa, Javier Trujillo, Manuel Monreal, for the RIETE investigators

**Affiliations:** 1 Hematology Service, Clínica Universidad de Navarra, IdISNA, Pamplona; CIBERCV, Instituto de Salud Carlos III, Madrid, Spain; 2 Deparment of Internal Medicine, Hospital Virgen de la Luz, Cuenca, Spain; 3 Deparment of Internal Medicine, Interdisciplinar Teragnosis and Radiosomics Research Group (INTRA-Madrid), Instituto de Salud Carlos III, University of Navarra, Clínica Universidad de Navarra, CIBERES, Madrid, Spain; 4 Department of Internal Medicine, Hospital Universitario de La Princesa, Madrid, Spain; 5 Department of Vascular Medicine, Ospedale Castelfranco Veneto, Castelfranco Veneto, Italy; 6 Department of Cardiology, Ospedale Pauls Stradins Clinical University Hospital, Riga Stradiņš University, Riga, Latvia; 7 Department of Emergency, Clermont-Ferrand University Hospital, Clermont-Ferrand, France; 8 Department of Internal Medicine, Hospital General Universitario Santa Lucía, Universidad Católica de Murcia, Murcia, Spain; 9 Department of Internal Medicine, Instituto de Salud Carlos III, Universidad Católica de Murcia, Hospital Germans Trias i Pujol, CIBERES, Badalona (Barcelona), Madrid, Spain

**Keywords:** venous thromboembolism, anticoagulation, bleeding, hemorrhage, score

## Abstract

The performance of validated bleeding risk scores in patients with venous thromboembolism (VTE) could be different depending on the time after index event or the site of bleeding. In this study we compared the “classic” Registro Informatizado de Enfermedad TromboEmbólica (RIETE) score and the more recently developed VTE-BLEED score for the prediction of major bleeding in patients under anticoagulant therapy in different time intervals after VTE diagnosis. Out of 82,239 patients with acute VTE, the proportion of high-risk patients according to the RIETE and VTE-BLEED scores was 7.1 and 62.3%, respectively. The performance of both scores across the different study periods (first 30 days after VTE diagnosis, days 31–90, days 91–180, and days 181–360) was similar, with areas under the receiving operating characteristics (ROC) curve (AUC) ranging between 0.69 and 0.72. However, the positive predictive values were low, ranging between 0.6 and 3.9 (better for early major bleeding than for later periods). A sensitivity analysis limited to patients with unprovoked VTE showed comparable results. Both scores showed a trend toward a better prediction of extracranial than intracranial major bleeding, the RIETE score resulting more useful for early extracranial bleeding and the VTE-BLEED for late intracranial hemorrhages. Our study reveals that the usefulness of available bleeding scores may vary depending on the characteristics of the patient population and the time frame evaluated. Dynamic scores could be more useful for this purpose.

## Introduction

Anticoagulant therapy is the mainstream of the management of venous thromboembolism (VTE). 1 Currently, several options are available: (1) initial parenteral therapy with unfractionated heparin, low-molecular weight heparin (LMWH), or fondaparinux followed by long-term oral vitamin K antagonists (VKAs); (2) LMWH for initial and long-term therapy (mostly used in cancer patients); and (3) direct oral anticoagulants (DOACs) alone or after an initial heparin lead-in period, depending on the drug. 2 Usually, the ideal length of treatment ranges between 3 months to indefinite depending on the estimated risk of recurrent VTE in case of discontinuing anticoagulation and the risk of bleeding associated with its maintenance. 3 Indeed, bleeding is the most common and severe adverse event related to anticoagulant drugs. Therefore, identification of patients at increased risk for bleeding is critical for decision-making.

In the latest years, different attempts to develop and validate a prognostic score to identify VTE patients at increased risk for bleeding have been performed, but their predictive values and accuracy are modest. 4 5 6 Recently, a new score to predict major bleeding in stable anticoagulated (i.e., after the first 30 days) patients with VTE, named VTE-BLEED score ( Table 1 ), was validated after a *post-hoc* analysis of data from two randomized clinical trials and a prospective cohort study comparing DOACs versus VKAs for the long-term treatment of VTE. 7 8 9 The long-term predictive ability of the score has also been confirmed in a retrospective study including consecutive patients with VTE, despite marked differences in the proportion of high-risk patients, 26 to 37% in the former studies versus 68% in the latter retrospective real-world registry. 10

**Table 1:** VTE-BLEED and RIETE scores

**VTE-BLEED**	**Score**
Active cancer	2
Male with uncontrolled arterial hypertension	1
Anemia a	1.5
History of bleeding	1.5
Age ≥60 y	1.5
Renal dysfunction (eGFR <60 mL/min)	1.5
*Risk categories*	
• *Low risk*	*<2 points*
• *High risk*	*≥2 points*
**RIETE**	**Score**
Recent major bleeding	2
Creatinine >1.2 mg/dL	1.5
Anemia a	1.5
Cancer	1
Clinically overt PE	1
Age >75 y	1
*Risk categories*	
• *Low risk*	*0 points*
• *Intermediate risk*	*1–4 points*
• *High risk*	*>4 points*

Abbreviations: eGFR, Estimated glomerular filtration rate; PE, pulmonary embolism; RIETE, Registro Informatizado de Enfermedad TromboEmbólica; VTE, venous thromboembolism.

a Anemia is defined as <13 g/dL in men and <12 g/dL in women.

The Registro Informatizado de Enfermedad TromboEmbólica (RIETE) is a multicenter, ongoing, international registry of consecutive patients with objectively confirmed, symptomatic acute VTE (ClinicalTrials.gov identifier: NCT02832245). Since its inception in 2001, the aim of RIETE is to record data including the clinical characteristics, treatment, and outcomes in patients diagnosed with VTE. 11 12 13 In the current study, we aimed to compare the predictive ability of the novel VTE-BLEED score with that of the previously developed RIETE score 14 ( Table 1 ) focusing on different time intervals after the index VTE event, also taking into account other relevant variables such as the site of the hemorrhage.

## Methods

### Patient Sample

The study population comprised consecutive patients enrolled in the RIETE registry between March 2001 and December 2019. The rationale and methodology have been already reported elsewhere. 11 12 13 Patients participating in a randomized trial with a blind medication were excluded. All suspected VTE events were objectively confirmed by compression ultrasound or contrast venography for deep vein thrombosis; helical computed tomography, or ventilation/perfusion scan or angiography for pulmonary embolism (PE). All patients or their family members provided written or oral consent for participation in the registry, in accordance with Local Ethics Committee's policies.

### Study Variables

The following parameters are recorded in the RIETE Registry: patients' demographics, comorbidities, risk factors for VTE, baseline laboratory data, and treatment received. In this study both, the RIETE bleeding score and the VTE-BLEED score for each patient were calculated. We defined *active cancer* when the diagnosis of a malignancy was made in 3 months previous to the VTE event, and in those patients, who presented with metastatic disease, or were receiving active therapy (chemotherapy, radiotherapy, hormonal therapy or palliative) at the time of VTE diagnosis. Skin malignancies were excluded. Uncontrolled hypertension was defined as values of systolic blood pressure levels >140 mm Hg at baseline.

### Outcomes

Our primary outcome was the risk for major bleeding, defined as any bleeding that was overt and required transfusion of two units or more of blood, or was retroperitoneal, spinal, or intracranial, or was fatal. 15 We compared the ability to predict major bleeding from both scores in patients receiving anticoagulant therapy at four different time periods: first 30 days, 31 to 90 days, 91 to 180 days, and 181 to 360 days.

### Statistical Analysis

The proportion of patients classified as low, intermediate, or high risk for bleeding is described. A comparison between the proportion of high-risk patients according to both scores was performed using the Fischer's exact test. The sensitivity, specificity, positive and negative predictive values, and likelihood ratios of both scores were estimated for the different time intervals. We evaluated the discriminative power of each score to predict major bleeding by calculating the area under the receiver operating characteristics (ROC) curve (AUC). Comparisons of the AUC derived from the same dataset were performed using the Hanley and McNeil method. 16 Sensitivity assessments in patients with unprovoked VTE, and according to the site of the bleeding (intracranial vs. extracranial) were performed. All calculations were done using IBM SPSS Statistics (version 20).

## Results

A total of 82,239 patients receiving anticoagulant therapy for acute VTE were included. Of these, 1,187 patients (1.4%) suffered a major bleeding event in the first 30 days after VTE diagnosis. Among patients who continued anticoagulant therapy 1 month after VTE diagnosis, 385 out 73,132 (0.5%) suffered a major bleeding event between days 31 and 90 after index VTE. The proportion of anticoagulated patients who developed a major bleeding between days 91 to 80 and days 181 to 360 was 243 out of 63,083 (0.4%) and 164 out of 35,685 patients (0.5%), respectively. The clinical characteristics of patients with major bleeding across the different study periods are depicted in Table 2 .

**Table 2:** Baseline characteristics of patients with or without major bleeding according to the time of occurrence

	*MB* *1–30 d*	*No MB* *1–30 d*	* MB a* *31–90 d*	* No MB a* *31–90 d*	*MB* b *91–180 d*	*No MB* b *91–180 d*	*MB* c *181–360 d*	*No MB* c *181–360 d*
*Patients, N*	*1,187*	*81,052*	*385*	*72,747*	*243*	*62,840*	*164*	*35,521*
*Clinical characteristics*								
Male gender	494 (42.0%)	39,761 (49.1%)	178 (46.2%)	35,669 (49.0%)	116 (47.7%)	30,953 (49.3%)	75 (45.7%)	17,695 (49.8%)
Age >85 y	204 (17.0%)	7,298 (9.0%)	58 (15.1%)	6,213 (8.5%)	44 (18.1%)	5,183 (8.2%)	24 (14.6%)	2,630 (7.4%)
Body weight <50 kg	57 (4.8%)	1,915 (2.4%)	15 (3.9%)	1,572 (2.2%)	8 (3.3%)	1,212 (1.9%)	5 (3.0%)	585 (1.6%)
Chronic lung disease	192 (16.0%)	9,370 (11.6%)	52 (13.5%)	8,261 (11.4%)	37 (15.2%)	6,994 (11.1%)	25 (15.2%)	3,925 (11.0%)
Chronic heart disease	140 (12.0%)	5,391 (6.7%)	42 (10.9%)	4,573 (6.3%)	15 (6.2%)	3,815 (6.1%)	23 (14.0%)	2,002 (5.6%)
History of stroke	67 (5.6%)	3,588 (4.4%)	22 (5.7%)	3,055 (4.2%)	18 (7.4%)	2,501 (4.0%)	17 (10.4%)	1,344 (3.8%)
History of hypertension ( *N* = 55.662)	436 (59%)	26,541 (48%)	142 (57%)	23,404 (48%)	98 (60%)	19,903 (48%)	71 (63%)	11,604 (49%)
*High risk for bleeding*								
Recent (<30 d) major bleeding	104 (8.8%)	1,720 (2.1%)	22 (5.7%)	1,343 (1.8%)	11 (4.5%)	1,020 (1.6%)	7 (4.3%)	435 (1.2%)
Active cancer	354 (30.0%)	14,500 (17.9%)	152 (39.5%)	11,686 (16.1%)	77 (31.7%)	8,588 (13.7%)	42 (25.6%)	4,032 (11.4%)
Anemia	671 (57.0%)	27,235 (33.6%)	215 (55.8%)	23,325 (32.1%)	134 (55.1%)	18,949 (30.2%)	93 (56.7%)	9,539 (26.9%)
Platelet count <50,000/µL	12 (1.0%)	290 (0.4%)	1 (0.3%)	178 (0.2%)	0 (0.0%)	123 (0.2%)	0 (0.0%)	51 (0.1%)
CrCl levels <30 mL/min	304 (26.0%)	8,028 (9.9%)	81 (21.0%)	6,636 (9.1%)	39 (16.0%)	5,484 (8.7%)	34 (20.7%)	2,873 (8.1%)
Antiplatelet use at VTE diagnosis	257 (22%)	11,879 (15%)	72 (19%)	10,386 (14%)	43 (18%)	8,804 (14%)	37 (23%)	4,799 (14%)
*VTE characteristics*								
PE (with or without DVT)	773 (65.0%)	42,613 (52.6%)	209 (54.3%)	37,432 (51.5%)	140 (57.6%)	32,554 (51.8%)	98 (59.8%)	20,043 (56.4%)
Proximal DVT	580 (49.0%)	44,246 (52.1%)	201 (52.2%)	38,396 (52.8%)	134 (55.1%)	33,396 (53.1%)	83 (50.6%)	18,859 (53.1%)
Distal DVT	75 (6.3%)	8,707 (10.7%)	26 (6.8%)	7,956 (10.9%)	20 (8.2%)	6,655 (10.6%)	13 (7.9%)	3,127 (8.8%)
Unprovoked	657 (55.0%)	53,874 (66.5%)	189 (49.1%)	49,470 (68.0%)	126 (51.9%)	44,249 (70.4%)	102 (62.2%)	26,210 (73.8%)
Surgery	178 (15.0%)	8,966 (11.1%)	36 (9.4%)	8,082 (11.1%)	30 (12.3%)	6,834 (10.9%)	13 (7.9%)	3,397 (9.6%)
Immobilization	414 (35.0%)	18,377 (22.7%)	118 (30.6%)	15,828 (21.7%)	72 (29.6%)	13,261 (21.1%)	46 (28.0%)	6,628 (18.7%)
Estrogen therapy	31 (2.6%)	4,416 (5.4%)	13 (3.4%)	4,050 (5.6%)	8 (3.3%)	3,592 (5.7%)	2 (1.2%)	1,964 (5.5%)
*Initial therapy*								
Low-molecular-weight heparin	959 (81.0%)	71,088 (87.7%)	353 (91.7%)	64,228 (88.3%)	216 (88.9%)	55,565 (88.4%)	143 (87.2%)	31,911 (89.8%)
Unfractionated heparin	127 (11.0%)	4,705 (5.8%)	17 (4.4%)	3,958 (5.4%)	17 (7.0%)	3,390 (5.4%)	12 (7.3%)	1,917 (5.4%)
Thrombolytics	60 (5.1%)	965 (1.2%)	2 (0.5%)	830 (1.1%)	2 (0.8%)	731 (1.2%)	1 (0.6%)	482 (1.4%)
DOACs	5 (0.4%)	1,975 (2.4%)	3 (0.8%)	1,884 (2.6%)	2 (0.8%)	1,614 (2.6%)	3 (1.8%)	579 (1.6%)
Fondaparinux	17 (1.4%)	1,573 (1.9%)	8 (2.1%)	1,376 (1.9%)	5 (2.1%)	1,126 (1.8%)	4 (2.4%)	411 (1.2%)
*Long-term therapy*								
Low-molecular-weight heparin	419 (35.0%)	22,534 (27.8%)	195 (50.6%)	18,855 (25.9%)	84 (34.6%)	12,963 (20.6%)	33 (20.1%)	4,776 (13.4%)
Anti-vitamin K	404 (34.0%)	49,136 (60.6%)	164 (42.6%)	47,791 (65.7%)	150 (61.7%)	44,690 (71.1%)	122 (74.4%)	28,570 (80.4%)
DOACs	20 (1.7%)	6,375 (7.9%)	21 (5.5%)	5,302 (7.3%)	7 (2.9%)	4,630 (7.4%)	6 (3.7%)	2,011 (5.7%)
*Bleeding location*								
Gastrointestinal tract	379 (31.9%)	–	153 (39.7%)	–	91 (37.4%)	–	69 (42.1%)	–
Genitourinary	114 (9.9%)	–	41 (10.6%)	–	22 (9.1%)	–	14 (8.5%)	–
Intracranial	134 (11.3%)	–	85 (22.1%)	–	72 (29.6%)	–	44 (26.8%)	–
Retroperitoneal	108 (9.1%)	–	22 (5.7%)	–	11 (4.5%)	–	8 (4.9%)	–
Muscle	136 (11.5%)	–	15 (3.9%)	–	11 (4.5%)	–	8 (4.9%)	–
Hematoma	194 (16.3%)	–	31 (8.1%)	–	19 (7.8%)	–	7 (4.3%)	–
Hemoptysis	32 (2.7%)	–	6 (1.6%)	–	7 (2.9%)	–	5 (3%)	–
Other	90 (7.6%)	–	32 (8.3%)	–	10 (4.2%)	–	9 (5.5%)	–

Abbreviations: DOAC, direct oral anticoagulant; DVT, deep vein thrombosis; PE, pulmonary embolism; VTE, venous thromboembolism.

a Patients on AC therapy by day 30.

b Patients on AC therapy by day 90.

c Patients on AC therapy by day 180.

### First 30 Days after VTE Diagnosis

The most frequent sites of the 1,187 major bleeds were gastrointestinal (31.9%), soft tissues (27.8%), and intracranial (11.3%). According to the RIETE score, 14,713 patients (17.9%) had a low risk for bleeding, 61,651 (75.0%) had intermediate risk, and 5,875 (7.1%) had high risk. Using the VTE-BLEED score, 30,974 patients (37.7%) were classified as low risk and 51,265 (62.3%) as high risk ( Table 3 ).

**Table 3:** Distribution of major bleeding events across risk categories and performance of each score during the studied periods

Days 1–30			Days 31–90 a			Days 91–180 b			Days 181–360 c		
Risk category	MB ( *N* = 1,187)	No MB ( *N* = 81,052)	Risk category	MB ( *N* = 385)	No MB ( *N* = 72,747)	Risk category	MB ( *N* = 243)	No MB ( *N* = 62,840)	Risk category	MB ( *N* = 164)	No MB ( *N* = 35,521)
RIETE low *N* = 14,713 (17.9%)	23 (0.2%)	14,690 (99.8%)	RIETE low *N* = 14,030 (19.2%)	13 (0.1%)	14,017 (99.9%)	RIETE low *N* = 12,653 (20.1%)	11 (0.1%)	12,642 (99.9%)	RIETE low *N* = 7,041 (19.7%)	6 (0.1%)	7,035 (99.9%)
RIETE interm. *N* = 61,651 (75.0%)	934 (1.5%)	60,717 (98.5%)	RIETE interm. *N* = 54,610 (74.7%)	312 (0.6%)	54,298 (99.4%)	RIETE interm. *N* = 46,941 (74.4%)	192 (0.4%)	46,749 (99.6%)	RIETE interm. *N* = 26,839 (75.2%)	127 (0.5%)	26,712 (99.5%)
RIETE high *N* = 5,875 (7.1%)	230 (3.9%) *	5,645 (96.1%)	RIETE high *N* = 4,492 (6.1%)	60 (1.3%) *	4,432 (98.7%)	RIETE high *N* = 3,489 (5.5%)	40 (1.1%) *	3,449 (98.9%)	RIETE high *N* = 1,805 (5.1%)	31 (1.7%) *	1,774 (98.3%)
VTE-Bleed low *N* = 30,974 (37.7%)	150 (0.5%)	30,824 (99.5%)	VTE-Bleed low *N* = 28,697 (39.2%)	46 (0.2%)	28,651 (99.8%)	VTE-Bleed low *N* = 25,743 (40.8%)	31 (0.1%)	25,712 (99.9%)	VTE-Bleed low *N* = 15,224 (42.7%)	32 (0.2%)	15,192 (99.8%)
VTE-Bleed high *N* = 51,265 (62.3%)	1,037 (2.0%) *	50,228 (98.0%)	VTE-Bleed high *N* = 44,435 (60.8%)	339 (0.8%) *	44,096 (99.2%)	VTE-Bleed high *N* = 37,340 (59.2%)	212 (0.6%) *	37,128 (99.4%)	VTE-Bleed high *N* = 20,461 (57.3%)	132 (0.6%) *	20,329 (99.4%)
	**VTE-Bleed**	**RIETE** **(High)**		**VTE-Bleed**	**RIETE** **(High)**		**VTE-Bleed**	**RIETE** **(High)**		**VTE-Bleed**	**RIETE** **(High)**
Sensitivity	87.4	19.4	Sensitivity	88.1	15.6	Sensitivity	87.2	16.5	Sensitivity	80.5	18.9
Specificity	38.0	93.0	Specificity	39.4	93.9	Specificity	40.9	94.5	Specificity	42.8	95.0
PPV	2.0	3.9	PPV	0.8	1.3	PPV	0.6	1.1	PPV	0.6	1.7
NPV	99.5	98.7	NPV	99.8	99.5	NPV	99.9	99.7	NPV	99.8	99.6
Accuracy	38.7	92.0	Accuracy	39.6	93.5	Accuracy	41.1	94.2	Accuracy	42.9	94.7
LR+	1.4	2.8	LR+	1.45	2.56	LR+	1.48	3.0	LR+	1.41	3.78
LR−	0.33	0.87	LR−	0.30	0.90	LR−	0.31	0.88	LR−	0.46	0.85

Abbreviations: AC, anticoagulant; Interm., intermediate; LR, likelihood ratio; MB, major bleeding; NPV, negative predictive value; PPV, positive predictive value; VTE, venous thromboembolism.

a Patients treated with AC >30 d.

b Patients treated with AC >90 d.

c Patients treated with AC >180 d.

* *p* < 0.001.

**Fig. 1:**
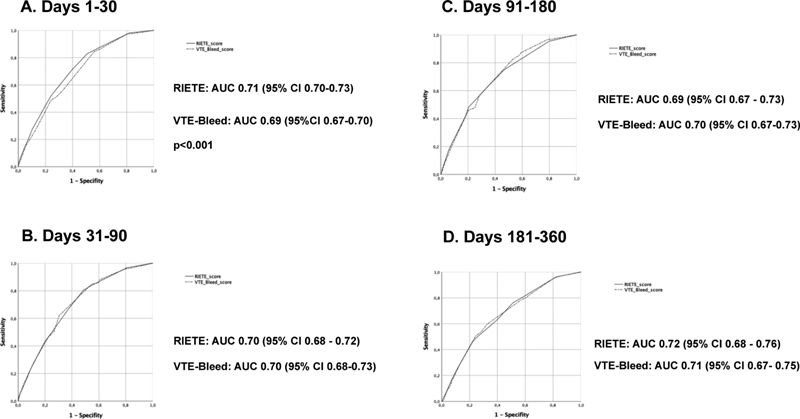
Receiver operating characteristic (ROC) curve for major bleeding in patients treated with anticoagulants.

The positive predictive values (PPVs) were 3.9% for the high-risk group of the RIETE score and 2.0% for the VTE-BLEED score, while the negative predictive values (NPVs) were 98.7 and 99.5%, respectively ( Table 3 ). The AUCs were 0.71 (95% confidence interval [CI] 0.70–0.73) and 0.69 (95% CI, 0.67–0.70), respectively ( *p* < 0.001) ( Fig. 1 ). Interestingly, differences in the AUC were mainly due to the superiority of the RIETE score for extracranial bleeding, while the performance on both scores was similar for intracranial bleeding ( Supplementary Fig. S1 ).

### Day 31 to Day 90 after VTE Diagnosis

During this study period, there were 385 major bleeding events. Again, gastrointestinal was the most frequent site (153 events; 39.7%) followed by intracranial bleeding (85; 22.1%). According to the RIETE score, 14,030 patients (19.2%) were classified as low risk for bleeding, 54,610 (74.7%) as intermediate risk, and 4,492 (6.1%) as high risk. Using the VTE-BLEED score, 28,697 patients (39.2%) were classified as low risk and 44,435 (60.8%) as high risk ( Table 3 ).

The PPVs of the high-risk strata were 1.3% for the RIETE score and 0.8% for the VTE-BLEED score, while the NPVs were 99.5 and 99.8%, respectively. The AUCs were almost identical: 0.70; (95% CI, 0.68–0.72) and 0.70 (95% CI, 0.68–0.73), respectively ( Fig. 1 ).

Similar results were obtained in a sensitivity analysis limited to patients with unprovoked VTE ( *N* = 49,659) ( Supplementary Table S1 and Supplementary Fig. S2 ). In this period both scores showed higher AUC for extracranial than for intracranial bleeding, without significant differences between them ( Supplementary Fig. S3 ).

### Day 91 to Day 180 after VTE Diagnosis

During this time interval, 243 major bleeding events were recorded. Again, gastrointestinal was the most frequent site (91 events; 37.4%) followed by intracranial (72 events; 29.6%). According to the RIETE score, 12,653 patients (20.1%) were classified as low risk for bleeding, 46,941 (74.4%) as intermediate risk, and 3,489 (5.5%) as high risk. Using the VTE-BLEED score, 25,743 patients (40.8%) were classified as low risk and 37,340 (59.2%) as high risk. The distribution of bleeding events across the different risk categories of each score and their performance is shown in Table 3 .

The PPVs were 2.3% for the RIETE score and 1.3% for the VTE-BLEED score, while the NPVs were 99.7 and 99.9%, respectively. The AUCs were similar: 0.69 (95% CI, 0.67–0.73) and 0.70 (95% CI, 0.67–0.73), respectively ( Fig. 1 ).

In a sensitivity analysis limited to patients with unprovoked VTE ( *N* = 44,375), similar findings were observed ( Supplementary Table S2 and Supplementary Fig. S4 ). Again, in this period both scores showed a trend toward higher AUC for extracranial than for intracranial bleeding, without significant differences between them ( Supplementary Fig. S5 ).

### Day 181 to Day 360 after VTE Diagnosis

Of the 164 major bleeding events recorded in this time interval, 69 (42.1%) were gastrointestinal, and 44 (26.8%) intracranial. According to the RIETE score, 7,041 patients (19.7%) were at low risk for bleeding, 26,839 (75.2%) at intermediate risk, and 1,805 (5.1%) at high risk. Using the VTE-BLEED score, 15,224 patients (42.7%) were classified as low risk and 20,461 (57.3%) as high risk ( Table 3 ).

The PPVs were 1.7% for the RIETE score and 0.6% for the VTE-BLEED score, while the NPVs were 99.6 and 99.8%, respectively. The AUCs were 0.72; (95% CI, 0.68–0.76) and 0.71 (95% CI, 0.67–0.75), respectively ( Fig. 1 ). Again, similar results were obtained in a sensitivity analysis limited to patients with unprovoked VTE ( *N* = 26,312) ( Supplementary Table S3 and Supplementary Fig. S6 ).

In this time interval the RIETE score showed a better AUC for extracranial bleeding than for intracranial bleeding, while the opposite trend was observed for the VTE-BLEED score. A trend toward a better performance of the RIETE score for extracranial bleeding and of the VTE-BLEED score for intracranial bleeding was noted ( Supplementary Fig. S7 ).

## Discussion

Although the RIETE and the VTE-BLEED scores share several common variables, we appreciate some differences in their performance. The RIETE score performed slightly better than the VTE-BLEED score for the evaluation of the risk within the first month of therapy. This was not unexpected since the VTE-BLEED score was derived to assess the risk for bleeding in patients under stable anticoagulation, at least 1 month after the index VTE event. 7 8 17 The possibility of presentation as PE (variable included in the RIETE score but not in the VTE-BLEED score) being a marker of early major bleeding cannot be discarded. However, a difference of 0.02 in the AUC may not be clinically relevant. Regarding later time intervals, despite the RIETE score was initially validated for the prediction of bleeding in the first 3 months, both scores performed rather similar, even in patients with unprovoked VTE. Indeed, this subgroup of patients is particularly relevant in clinical practice since most guidelines recommend the use of indefinite anticoagulation if the risk of bleeding is not high. 1 3

Accurate tools for the evaluation of the bleeding risk during the course of anticoagulant therapy for VTE are needed. In the short-term, high-risk patients could benefit from a narrower surveillance and selection of drugs with a better safety profile. 18 In the long-term, the risk assessment should be considered to decide the duration of anticoagulant therapy. 19 Our study suggests that the usefulness of available bleeding scores may vary depending on the characteristics of the patient population and the time frame evaluated. In this real-world population we confirm that the proportion of patients with VTE classified at high-risk using the VTE-BLEED score is much higher than that initially found in the randomized clinical trials that led to derivation and validation of the score (62% in our series vs. 25–35%). 10 According to the RIETE score, 75% of the patients were allocated to the intermediate risk category and 7% to the high-risk stratum.

Another interesting finding of the present study is that the predictive ability of the two scores may also vary according to the site of bleeding. Both, the RIETE and VTE-BLEED scores showed higher AUC for extracranial than for intracranial hemorrhages (ICHs) during the first 6 months. On the contrary, for later bleeding events, the AUC of the VTE-BLEED score was better for ICH than for extracranial bleeding. In fact, the better performance of the RIETE score for early bleeding was associated mainly with extracranial bleedings, while late (beyond the first 6 months) ICH was better predicted by the VTE-BLEED score. A possible explanation is that the variable uncontrolled hypertension is not included in the RIETE score. On the other hand, extracranial hemorrhages occur more often in the first days after VTE diagnosis compared with ICH. 20 In a recent sub-analysis of the Hokusai-VTE and Recover trials, the pooled odds ratio of the VTE-BLEED score for predicting ICH or fatal bleeding was 4.7 (95% CI 2.2–10), although the incidence of the outcome was low. 21 Similarly, in our series, the OR of the VTE-BLEED score for ICH between day 31 and day 180 was 4.2 (data not shown).

It could be argued that the performance of the RIETE score might have been overestimated, due to the inclusion of the population from which it was developed. However, the current study includes more than 82,000 patients while the original report included 19,000 patients and was limited to the first 3 months after index VTE. Similar results were observed if the analysis was limited to patients registered in RIETE after 2010 (data not shown). The higher number of patients and events in the current study is a strength to take into account regarding other previous studies that have compared bleeding scores in VTE patients, in which the c-statistic of the RIETE score was more modest. 6 22 In another recent prospective study, the AUC of the RIETE and VTE-BLEED scores for the detection of in-hospital bleeding in patients with acute PE were also high: 0.77 and 0.75, respectively. The addition of D-dimer values could help to improve their performance. 23

Despite the results highlighted by this study, both scores have a suboptimal predictive ability, particularly their PPV is poor. Their usefulness should be tested in appropriately designed clinical trials, for example as decision tools for prolongation or withdrawal of anticoagulant therapy in patients with unprovoked VTE after completion of 3 to 6 months of treatment. Our results open the debate about the need of different scores depending on the time frame evaluated, what would imply a more complex scenario.

Several limitations of the study are acknowledged. First, the use of a single baseline evaluation for the assessment of delayed bleeding risk is controversial. Probably, for decisions on extension of anticoagulant therapy periodical evaluations are required. Dynamic scores, not available yet, could be more useful for this purpose. Second, most patients in our registry received long-term therapy with VKAs. We lack reliable data about the quality of INR monitoring. This data could be particularly valuable for the evaluation of early bleeding, sometimes related with the transition from LMWH to VKAs. In addition, the HAS-BLED and Seiler's scores could not be included in the evaluation, since we lacked information for the item “labile INR” 24 25 . Finally, the number of patients receiving treatment with DOACs is very low. A different behavior of a score in a population of patients uniformly treated with these drugs cannot be ruled out.

In conclusion, the RIETE and the VTE-BLEED score performed similarly for the prediction of early and late bleeds, with small differences depending on the time since VTE diagnosis and the site of hemorrhage.

## Notes

Appendix

**Coordinator of the RIETE Registry:** Manuel Monreal.
**RIETE Steering Committee Members:** Paolo Prandoni, Benjamin Brenner, and Dominique Farge-Bancel.
**RIETE National Coordinators:** Raquel Barba (Spain), Pierpaolo Di Micco (Italy), Laurent Bertoletti (France), Sebastian Schellong (Germany), Inna Tzoran (Israel), Abilio Reis (Portugal), Marijan Bosevski (R. Macedonia), Henri Bounameaux (Switzerland), Radovan Malý (Czech Republic), Peter Verhamme (Belgium), Joseph A. Caprini (United States), Hanh My Bui (Vietnam).
**RIETE Registry Coordinating Center:** S & H Medical Science Service.

**Members of the RIETE Group**
**Spain:** Adarraga MD, Aibar J, Aibar MA, Alonso J, Amado C, Arcelus JI, Asuero A, Ballaz A, Barba R, Barbagelata C, Barrón M, Barrón-Andrés B, Blanco-Molina A, Botella E, Camon AM, Casado I, Castro J, Chasco L, Criado J, de Ancos C, del Toro J, Demelo-Rodríguez P, Díaz-Brasero AM, Díaz-Peromingo JA, Di Campli MV, Dubois-Silva A, Escribano JC, Falgá C, Farfán-Sedano AI, Fernández-Capitán C, Fernández-Reyes JL, Fidalgo MA, Flores K, Font C, Font L, Francisco I, Gabara C, Galeano-Valle F, García MA, García-Bragado F, García de Herreros M, García de la Garza R, García-Díaz C, García-Hernáez R, García-Raso A, Gil-Díaz A, Giménez-Suau M, Gómez-Cuervo C, Grau E, Guirado L, Gutiérrez J, Hernández-Blasco L, Hernando E, Jara-Palomares L, Jaras MJ, Jiménez D, Jiménez R, Jiménez-Alfaro C, Joya MD, Laguna JC, Latorre A, Lecumberri R, Lima J, Llamas P, Lobo JL, López-Jiménez L, López-Miguel P, López-Núñez JJ, López-Reyes R, López-Sáez JB, Lorenzo A, Madridano O, Maestre A, Marchena PJ, Martín-Martos F, Martínez-Urbistondo D, Mella C, Mercado MI, Moisés J, Monreal M, Muñoz M, Muñoz-Blanco A, Muñoz-Rivas N, Navas MS, Nieto JA, Nofuentes-Pérez E, Núñez-Fernández MJ, Obispo B, Olid M, Olivares MC, Orcastegui JL, Osorio J, Otalora S, Otero R, Parra P, Pedrajas JM, Pellejero G, Porras JA, Portillo J, Rivera-Civico F, Rodríguez-Chiaradía DA, Rodríguez-Matute C, Rogado J, Rosa V, Ruiz-Artacho P, Ruiz-Giménez N, Ruiz-Ruiz J, Ruiz-Sada P, Salgueiro G, Sánchez-Martínez R, Sánchez-Muñoz-Torrero JF, Sancho T, Soler S, Suárez-Rodríguez B, Suriñach JM, Tirado R, Torres MI, Tolosa C, Trujillo-Santos J, Uresandi F, Valero B, Valle R, Vela JR, Vela L, Vidal G, Villalobos A, Villares P, Zamora C, **Argentina:** Gutiérrez P, Vázquez FJ, **Belgium:** Engelen M, Vandenbriele C, Verhamme P, **Czech Republic** : Hirmerova J, Malý R, **France:** Ait Abdallah N, Bertoletti L, Bura-Riviere A, Catella J, Crichi B, Debourdeau P, Espitia O, Falvo N, Farge-Bancel D, Helfer H, Mahé I, Moustafa F, Poenou G, Quere I, **Germany:** Schellong S, **Israel:** Braester A, Brenner B, Tzoran I, **Iran:** Nikandish R, **Italy:** Bilora F, Brandolin B, Bucherini E, Ciammaichella M, Di Micco P, Imbalzano E, Maida R, Pace F, Pesavento R, Prandoni P, Quintavalla R, Rocci A, Siniscalchi C, Tufano A, Visonà A, Zalunardo B, **Latvia:** Make K, Skride A, Strautmane S, **Portugal:** Fonseca S, Martins F, Meireles J, **Republic of Macedonia:** Bosevski M, **Switzerland** : Bounameaux H, Mazzolai L, **USA:** Ochoa-Chaar CI, Weinberg I, **Vietnam** : Bui HM.
