# Supplementary material for: Prediction of Major Bleeding in Anticoagulated Patients for Venous Thromboembolism: Comparison of the RIETE and the VTE-BLEED Scores
Source: TH Open. 2021 Aug 9;5(3):e319–28. doi: 10.1055/s-0041-1729171 (PMC8459175; doi:10.1055/s-0041-1729171)
Supplement: Supplementary file 1 — Supplementary Material [file 10-1055-s-0041-1729171-s210013.pdf]

Supplementary Material

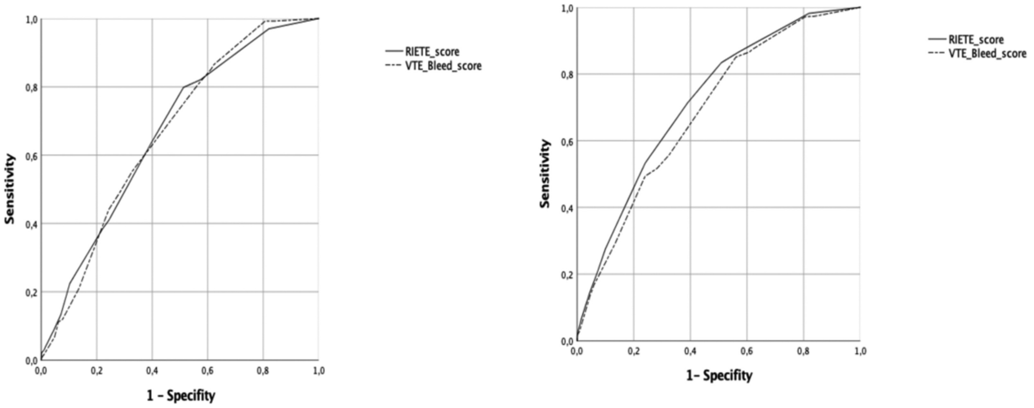

Supplementary Fig. S1 AUC for major bleeding (days 1–30) according to location of the hemorrhage. AUC, area under the receiving operating characteristics curve; ECH, extracranial hemorrhage; ICH, intracranial hemorrhage; RIETE, Registro Informatizado de Enfermedad TromboEmbólica; VTE, venous thromboembolism. ICH (1–30 days) ECH (1–30 days). RIETE: AUC 0.67 (95% CI 0.63–0.71), RIETE: AUC 0.72 (95% CI 0.71–0.73). VTE-Bleed: AUC 0.66 (95% CI 0.63–0.70), VTE-Bleed: AUC 0.69 (95% CI 0.67–0.70). $p < 0.001$.

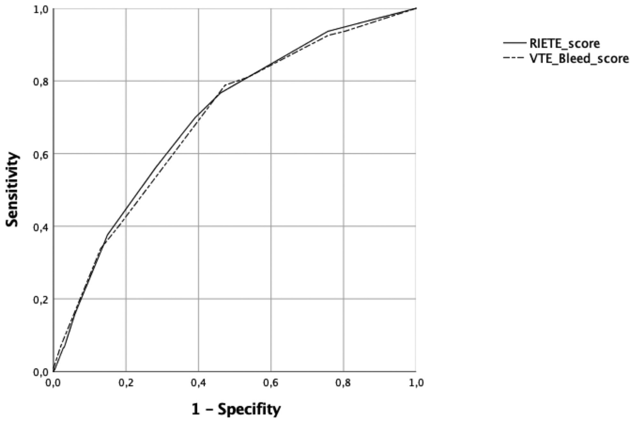

Supplementary Fig. S2 AUC for major bleeding (days 31–90) in patients treated with AC with unprovoked VTE. AC, anticoagulant; AUC, area under the receiving operating characteristics curve; RIETE, Registro Informatizado de Enfermedad TromboEmbólica; VTE, venous thromboembolism. RIETE: AUC 0.70 (95% CI 0.66–0.73). VTE-Bleed: AUC 0.69 (95% CI 0.66–0.73).

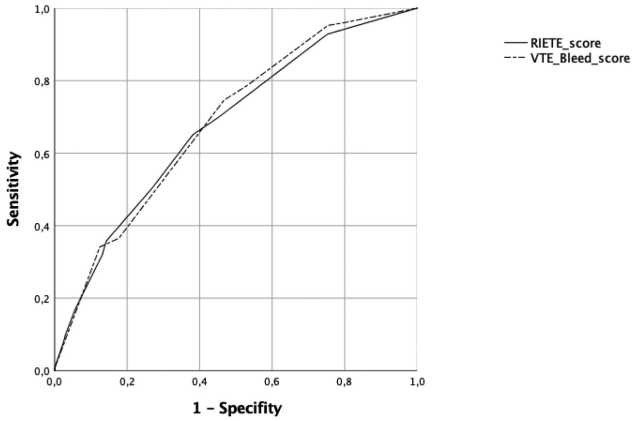

Supplementary Fig. S4 AUC for major bleeding (days 91–180) in patients treated with AC with unprovoked VTE. AC, anticoagulant; AUC, area under the receiving operating characteristics curve; RIETE, Registro Informatizado de Enfermedad TromboEmbólica; VTE, venous thromboembolism. RIETE: AUC 0.68 (95% CI 0.63–0.72). VTE-Bleed: AUC 0.68 (95% CI 0.64–0.73).

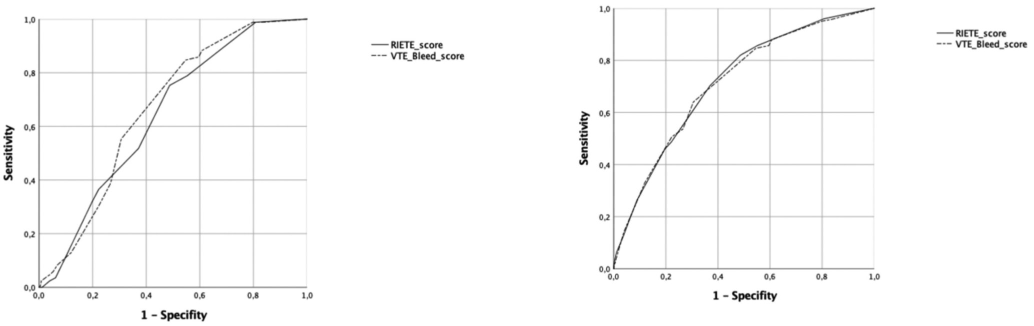

Supplementary Fig. S3 AUC for major bleeding (days 31–90) in patients treated with anticoagulants more than 30 days, according to location of the hemorrhage. AUC, area under the receiving operating characteristics curve; ECH, extracranial hemorrhage; ICH, intracranial hemorrhage; RIETE, Registro Informatizado de Enfermedad TromboEmbólica; VTE, venous thromboembolism. ICH (31–90 days) ECH (31–90 days). RIETE: AUC 0.64 (95% CI 0.59–0.69) RIETE: AUC 0.72 (95% CI 0.69–0.74). VTE-Bleed: AUC 0.66 (95% CI 0.62–0.71) VTE-Bleed: AUC 0.71 (95% CI 0.69–0.74).

 © 2021. The Author(s).

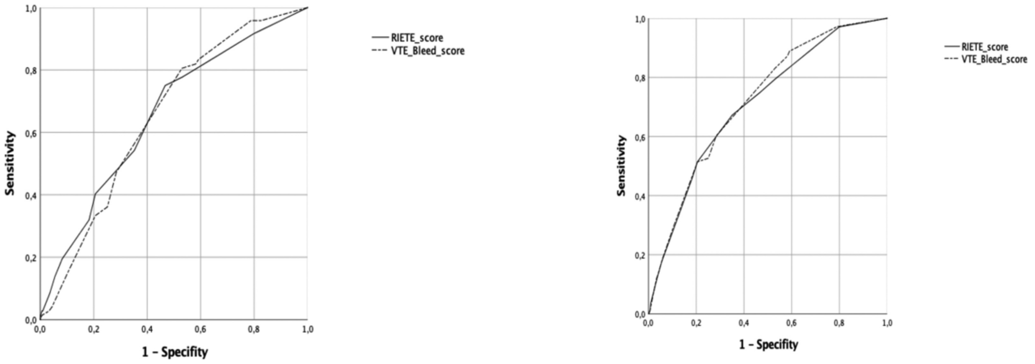

Supplementary Fig. S5 AUC for major bleeding (days 91–180) in patients treated with anticoagulants more than 90 days, according to location of the hemorrhage. AUC, area under the receiving operating characteristics curve; ECH, extracranial hemorrhage; ICH, intracranial hemorrhage; RIETE, Registro Informatizado de Enfermedad TromboEmbólica; VTE, venous thromboembolism. ICH (91–180 days) ECH (91–180 days). RIETE: AUC 0.65 (95% CI 0.59–0.72) RIETE: AUC 0.71 (95% CI 0.67–0.75). VTE-Bleed: AUC 0.65 (95% CI 0.60–0.70) VTE-Bleed: AUC 0.72 (95% CI 0.69–0.77).

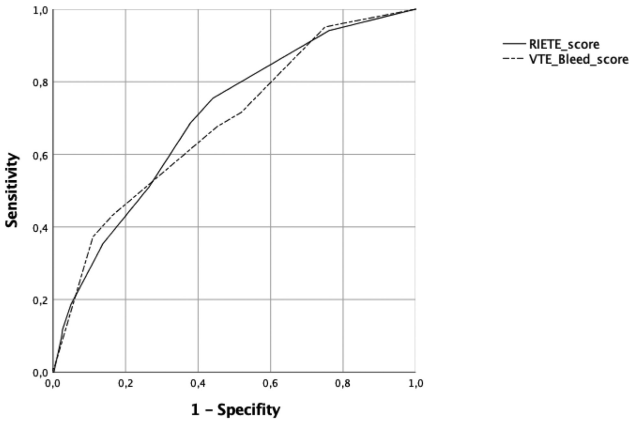

Supplementary Fig. S6 AUC for major bleeding (days 181–360) in patients with unprovoked VTE treated with AC more than 180 days. AC, anticoagulant; AUC, area under the receiving operating characteristics curve; RIETE, Registro Informatizado de Enfermedad TromboEmbólica; VTE, venous thromboembolism. RIETE: AUC 0.70 (95% CI 0.65–0.75). VTE-Bleed: AUC 0.69 (95% CI 0.63–0.74).

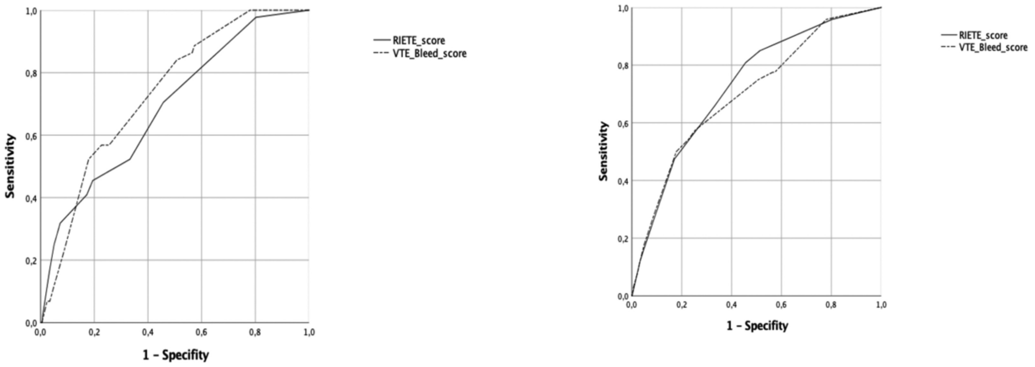

Supplementary Fig. S7 AUC for major bleeding (days 181–360) in patients treated with AC more than 180 days, according to location of the hemorrhage. AUC, area under the receiving operating characteristics curve; AC, anticoagulant; ECH, extracranial hemorrhage; ICH, intracranial hemorrhage; RIETE, Registro Informatizado de Enfermedad TromboEmbólica; VTE, venous thromboembolism. ICH (181–360 days) ECH (181–360 days). RIETE: AUC 0.69 (95% CI 0.61–0.77) RIETE: AUC 0.73 (95% CI 0.69–0.77). VTE-Bleed: AUC 0.74 (95% CI 0.67–0.80) VTE-Bleed: AUC 0.70 (95% CI 0.65–0.75).

 © 2021. The Author(s).

Supplementary Table S1 Sensitivity analysis for major bleeding (days 31–90) in patients treated with AC with unprovoked VTE

Patients treated with AC >30 d	MB ($N = 189$)	No MB ($N = 49,470$)
RIETE low risk $N = 12,058$ (24.3%)	12 (0.1%)	12,046 (99.9%)
RIETE intermediate risk $N = 36,078$ (72.7%)	164 (0.5%)	35,914 (99.5%)
RIETE high risk $N = 1,523$ (3.1%)	13 (0.9%)*	1,510 (99.1%)
VTE-BLEED low risk $N = 23,064$ (46.4%)	36 (0.2%)	23,028 (99.8%)
VTE-BLEED high risk $N = 26,595$ (53.6%)	153 (0.6%)*	26,442 (99.4%)
	VTE-Bleed	RIETE (High-risk)
Sensitivity	81.0	6.9
Specificity	46.5	96.9
PPV	0.6	0.9
NPV	99.8	99.6
Accuracy	46.7	96.6
LR+	1.51	2.25
LR−	0.41	0.96

Abbreviations: AC, anticoagulant; MB, major bleeding; LR, likelihood ratio; NPV, negative predictive value; PPV, positive predictive value; RIETE, Registro Informatizado de Enfermedad TromboEmbólica; VTE, venous thromboembolism.
*$p < 0.05$.

Supplementary Table S2 Sensitivity analysis for major bleeding (days 91–180) in patients treated with AC with unprovoked VTE

Patients treated with AC >90 d	MB ($N = 126$)	No MB ($N = 44,249$)
RIETE low risk $N = 10,943$ (24.7%)	9 (0.1%)	10,934 (99.9%)
RIETE intermediate risk $N = 32,155$ (72.5%)	105 (0.3%)	32,050 (99.7%)
RIETE high risk $N = 1,277$ (2.9%)	12 (0.9%)*	1,265 (99.1%)
VTE-BLEED low risk $N = 20,834$ (46.9%)	27 (0.1%)	20,807 (99.9%)
VTE-BLEED high risk $N = 23,541$ (53.1%)	99 (0.4%)*	23,442 (99.6%)
	VTE-Bleed	RIETE (High risk)
Sensitivity	78.6	9.5
Specificity	47.0	97.1
PPV	0.4	0.9
NPV	99.9	99.7
Accuracy	47.1	96.9
LR+	1.48	3.33
LR-	0.46	0.93

Abbreviations: AC, anticoagulant; MB, major bleeding; LR, likelihood ratio; NPV, negative predictive value; PPV, positive predictive value; RIETE, Registro Informatizado de Enfermedad TromboEmbólica; VTE, venous thromboembolism.
*$p < 0.05$.

Supplementary Table S3 Sensitivity analysis for major bleeding (days 181–360) in patients with unprovoked VTE treated with AC more than 180 days

Patients treated with AC >180 d	MB ($N = 102$)	No MB ($N = 26,210$)
RIETE low risk $N = 6,252$(23.8%)	6 (0.1%)	6,246 (99.9%)
RIETE intermediate risk $N = 19,361$ (73.6%)	84 (0.4%)	19,277 (99.6%)
RIETE high risk $N = 699$ (2.7%)	12 (1.7%)*	687 (98.3%)
VTE-BLEED low risk $N = 12,635$ (48.0%)	29 (0.2%)	12,606 (99.8%)
VTE-BLEED high risk $N = 13,677$ (52.0%)	73 (0.5%)*	13,604 (99.5%)
	VTE-Bleed	RIETE (High risk)
Sensitivity	71.6	11.8
Specificity	48.1	97.4
PPV	0.5	1.7
NPV	99.8	99.6
Accuracy	48.2	97.0
LR+	1.38	4.49
LR-	0.59	0.91

Abbreviations: AC, anticoagulant; MB, major bleeding; LR, likelihood ratio; NPV, negative predictive value; PPV, positive predictive value; RIETE, Registro Informatizado de Enfermedad TromboEmbólica; VTE, venous thromboembolism.
*$p < 0.001$.

 © 2021. The Author(s).
